# Leptin deficiency-induced obesity affects the density of mast cells in abdominal fat depots and lymph nodes in mice

**DOI:** 10.1186/1476-511X-11-21

**Published:** 2012-02-07

**Authors:** Mehmet M Altintas, Behzad Nayer, Eric C Walford, Kevin B Johnson, Gabriel Gaidosh, Jochen Reiser, Nestor De La Cruz-Munoz, Luis M Ortega, Ali Nayer

**Affiliations:** 1 Department of Medicine, Miller School of Medicine, University of Miami, Miami, FL, USA; 2 Diabetes Research Institute, University of Miami, Miami, FL, USA; 3 Bascom Palmer Eye Institute, Department of Ophthalmology, University of Miami, Coral Gables, FL, USA; 4 College of Engineering, University of Miami, Coral Gables, FL, USA; 5 Department of Surgery, Miller School of Medicine, University of Miami, Miami, FL, USA

**Keywords:** Obesity, leptin, mast cells, adipose tissue, inflammation

## Abstract

**Background:**

Mast cells are implicated in the pathogenesis of obesity and insulin resistance. Here, we explored the effects of leptin deficiency-induced obesity on the density of mast cells in metabolic (abdominal fat depots, skeletal muscle, and liver) and lymphatic (abdominal lymph nodes, spleen, and thymus) organs. Fourteen-week-old male leptin-deficient *ob/ob *mice and their controls fed a standard chow were studied. Tissue sections were stained with toluidine blue to determine the density of mast cells. CD117/c-kit protein expression analysis was also carried out. Furthermore, mast cells containing immunoreactive tumor necrosis factor-α (TNF-α), a proinflammatory cytokine involved in obesity-linked insulin resistance, were identified by immunostaining.

**Results:**

*ob/ob *mice demonstrated adiposity and insulin resistance. In abdominal fat depots, mast cells were distributed differentially. While most prevalent in subcutaneous fat in controls, mast cells were most abundant in epididymal fat in *ob/ob *mice. Leptin deficiency-induced obesity was accompanied by a 20-fold increase in the density of mast cells in epididymal fat, but a 13-fold decrease in subcutaneous fat. This finding was confirmed by CD117/c-kit protein expression analysis. Furthermore, we found that a subset of mast cells in epididymal and subcutaneous fat were immunoreactive for TNF-α. The proportion of mast cells immunoreactive for TNF-α was higher in epididymal than in subcutaneous fat in both *ob/ob *and control mice. Mast cells were also distributed differentially in retroperitoneal, mesenteric, and inguinal lymph nodes. In both *ob/ob *mice and lean controls, mast cells were more prevalent in retroperitoneal than in mesenteric and inguinal lymph nodes. Leptin deficiency-induced obesity was accompanied by increased mast cell density in all lymph node stations examined. No significant difference in the density of mast cells in skeletal muscle, liver, spleen, and thymus was noted between *ob/ob *and control mice.

**Conclusions:**

This study demonstrates that leptin deficiency-induced obesity is accompanied by alterations in the density of mast cells in abdominal fat depots. The divergent distribution of mast cells in subcutaneous versus visceral fat might partially account for their differential biological behavior. Mast cells might also play a role in adaptive immune response occurring in regional lymph nodes in obesity.

## Background

Obesity has reached epidemic proportions in many parts of the world [1]. Obesity if often accompanied by a low-grade systemic inflammatory state and adipose tissue inflammation [2]. Although the underlying mechanisms that induce adipose tissue inflammation in obesity remain largely elusive, adipocyte injury and death appear to play a central role [3]. Cells and mediators of both innate and adaptive immunity are involved in adipose tissue inflammation in obesity. In obese rodents and humans, monocytes infiltrate adipose tissue and differentiate into proinflammatory macrophages [4]. In addition, subsets of T lymphocytes, including regulatory T cells [5], CD8^+ ^effector T cells [6], and natural killer T cells [7], are involved in adipose tissue inflammation in obesity.

In addition to their role in host defense, mast cells have been implicated in a variety of inflammatory and autoimmune diseases, such as allergic reactions, bullous pemphigoid, multiple sclerosis, inflammatory arthritis, and atherosclerosis [8]. Mast cells accumulate in the adipose tissue of obese human subjects and diet-induced obese mice [9-11]. Furthermore, mast cell deficiency and mast cell stabilizers are shown to diminish adverse metabolic effects of a high-fat diet [10]. Of note, we have shown that mast cells in the epididymal fat of diet-induced obese mice contain and secrete tumor necrosis factor-α (TNF-α), a proinflammatory cytokine implicated in the pathogenesis of obesity [11].

There is a remarkable diversity in the structure and function of the adipose tissue found in different anatomical locations [12,13]. Whereas visceral adiposity is closely associated with adverse cardiovascular outcome, increased subcutaneous fat, especially around thighs and hips, poses little to no risk [14]. We recently demonstrated that macrophages and mast cells are distributed differentially in abdominal fat depots of both lean and diet-induced obese mice [11]. We also showed that diet-induced obesity in mice is associated with a marked increase in mast cells in the visceral, but not in the subcutaneous, fat depots [11]. Although adipose tissue inflammation in obesity and its metabolic sequelae have been the focus of intense research over the past two decades, little is known about immune responses that take place in regional lymph nodes draining inflamed adipose tissues. Lymph nodes are strategically located lymphoid tissues where innate immune responses result in adaptive immunity [15]. The involvement of lymphocytes in adipose tissue inflammation in obesity suggests a crosstalk between innate and adaptive immune systems in peripheral lymphoid tissues. Although there are many potential targets, we examined the effects of adipose tissue inflammation on mast cells in abdominal lymph nodes.

The present study explored whether obesity and insulin resistance as a result of leptin deficiency affect the density and distribution of mast cells in metabolic (abdominal fat depots, skeletal muscle, and liver) and lymphatic (abdominal lymph nodes, spleen, and thymus) organs. We also determined the prevalence of mast cells immunoreactive for TNF-α in epididymal and inguinal (subcutaneous) fat depots.

## Methods

### Experimental animals

We followed the 'Principles of laboratory animal care' established by the National Institutes of Health. The Institutional Animal Care and Use Committee of the University of Miami approved all procedures on experimental animals (IACUC protocol number 08-245). Male leptin-deficient (*ob/ob*, n = 10) and control (*+/?*, n = 10) mice were purchased from the Jackson Laboratory (Bar Harbor, ME) and acclimated for three weeks before blood and tissue sampling was carried out at the age of 14 weeks. Mice were fed on a standard chow (Rodent Diet 5010, LabDiet, St. Louis, MO). Mice were weighed with a Scout Pro balance SP202 (Ohaus, Pine Brook, NJ). Organ weights were measured with a Sartorius ED124S Analytical Balance (Sartorius, Bohemia, NY).

### Glucose, insulin and cholesterol assays

Blood was obtained from the tail of unanesthetized mice after overnight fasting for 15 hours. Blood glucose concentrations were measured using a Contour glucometer (Bayer, Tarrytown, NY). Serum insulin concentrations were measured by immunoassay following manufacturer's instructions (Crystal Chem, Downer Grover, IL). Serum cholesterol concentrations were determined using Cholesterol LiquiColor kit (Stanbio Laboratory, Boerne, TX). Homeostasis Model of Assessment-Insulin Resistance (HOMA-IR) was calculated using the formula: fasting glucose (mg/dl) × fasting insulin (mU/L)/405.

### Harvesting abdominal fat depots and lymph nodes

**Figure 1:**
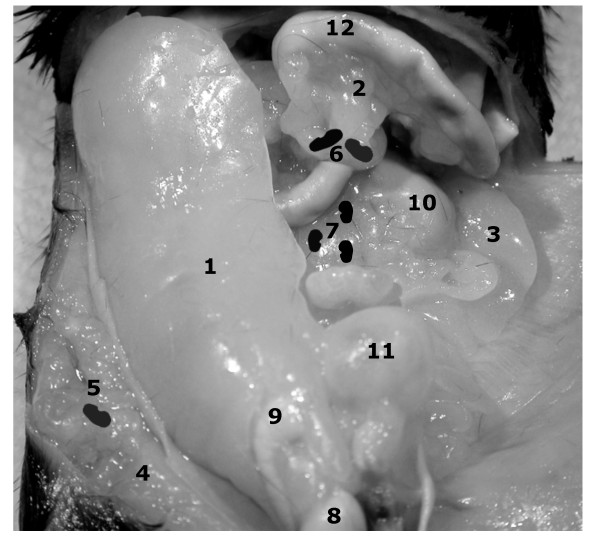
**Abdominal fat depots and lymph nodes in the mouse**. The epididymal (1), mesenteric (2), perinephric (3), and inguinal (4) fat depots of a male *ob/ob *mouse are demonstrated. Furthermore, the inguinal (5), mesenteric (6), and retroperitoneal (7) lymph nodes are represented schematically. The right testis (8), right epididymis (9), left kidney (10), urinary bladder (11), and a small bowel loop (12) are also shown.

The anatomy of abdominal fat depots and lymph nodes are illustrated in Figure 1. Non-fasted mice were euthanized and partially skinned. The right inguinal fat depot harboring the inguinal lymph node was dissected. After harvesting the right epididymal fat depot, the attached testis and epididymis were dissected under a Nikon SMZ 1500 microscope (Nikon, Melville, NY). The small intestine, attached mesenteric fat, and mesenteric lymph nodes were removed in toto. The retroperitoneal organs, including kidneys, retroperitoneal fat depots and retroperitoneal lymph nodes, were dissected off posterior abdominal wall.

### Quantification of mast cells

Tissues were fixed in Carnoy's solution and embedded in paraffin. Five micron-thick sections were cut, baked at 60°C for one hour, deparaffinized in xylene, and rehydrated in a graded ethanol series and water. To demonstrate mast cells, toluidine blue staining was carried out by briefly submerging tissue sections in 0.1% aqueous toluidine blue (Electron Microscopy Sciences, EMS, Hatfield, PA) [11]. Mast cells were counted in twenty high-power fields (400X) of toluidine blue-stained sections and their density was expressed as mast cells per mm^2 ^of tissue section. In the epididymal fat, mast cells were counted along the long axis of the fat depot from rostral to caudal. In the mesenteric, perinephric, and inguinal subcutaneous fat depots, mast cells were counted in areas away from the mesenteric, retroperitoneal, and inguinal lymph nodes, respectively.

### Enzyme histochemistry

Esterase activity of mast cells was demonstrated as previously described [11]. Briefly, tissue sections were stained with a mixture of new fuchsin acid solution (Poly Scientific, Bay Shore, NY), 4% sodium nitrite solution (Sigma-Aldrich, St. Louis, MO), and naphthol AS-D chloroacetate solution (Sigma-Aldrich) in 0.1 M sodium phosphate buffer, pH 7.6 (EMS) and counterstained with Mayer's hematoxylin (EMS). Light microscopic images were acquired using a Leica DMLB microscope with a Leica DFC420 C color camera (Leica, Bannockburn, IL).

### Immunofluorescence staining

After blocking with 5% goat serum, deparaffinized tissue sections were sequentially incubated with polyclonal rabbit anti-mouse TNF-α antibody (1:250; Abcam, Cambridge, MA) and Alexa Fluor 594-labeled goat anti-rabbit IgG (1:1000; Molecular Probes, Eugene, OR). Mast cells were labeled with FITC-conjugated avidin (1:100; BD Pharmingen, San Jose, CA) as previously described [11,16].

### Protein extraction and immunoblotting

Epididymal and inguinal adipose tissue (100 mg) were homogenized in 0.5 ml ice-cold lysis buffer containing 50 mM Tris-HCl (pH 7.4), 150 mM NaCl, 1% NP-40, 0.5% sodium deoxycholate, 0.1% SDS, 5 mM EDTA, 1 mM EGTA (Boston BioProducts, Ashland, MA) supplemented with a cocktail of protease inhibitors (Complete Mini, Roche Applied Science, Indianapolis, IN). Following agitation on a rotator for 1 hour and centrifugation at 13,000 rpm for 20 minutes at 4°C, middle phase of lysate containing 30 μg protein was subjected to polyacrylamide gel electrophoresis (NuPAGE Bis-Tris system, Invitrogen, Carlsbad, CA). A rabbit polyclonal antibody against CD117/c-kit (1:1000, Dako, Carpinteria, CA) and a mouse monoclonal antibody against actin (1:1000, Sigma-Aldrich) were used for immunoblotting.

### Statistics

Results are presented as mean ± SEM. Unpaired Student's t test was used to assess for statistically significant differences between groups. Comparisons among multiple groups were made using one-way analysis of variance (ANOVA) with Tukey post-hoc analysis. GraphPad Prism software (5.0a) was used for calculations (GraphPad Software, La Jolla, CA).

## Results

### Leptin-deficient *ob/ob *mice demonstrated adiposity, insulin resistance, and hypercholesterolemia

Compared to controls, *ob/ob *mice demonstrated greater body mass, fasting blood glucose, serum insulin and cholesterol concentrations, and HOMA-IR (Homeostatic model assessment-insulin resistance) (Table 1). In addition, *ob/ob *mice had larger liver, inguinal and epididymal fat depots than control mice (Table 1).

**Table 1:** Metabolic characteristics of mice

Variables	*ob/ob *(n = 10)	*+/? *(n = 10)	*P *value
Body weight (g)	60.2 ± 1.2	33.0 ± 0.7	< 0.001

Epididymal fat weight (g)	1.70 ± 0.04	0.41 ± 0.05	< 0.001

Inguinal fat weight (g)	1.86 ± 0.07	0.21 ± 0.02	< 0.001

Liver weight (g)	4.96 ± 0.17	1.53 ± 0.06	< 0.001

Blood glucose (mg/dl)	115 ± 8	64 ± 3	< 0.001

Serum insulin (ng/ml)	5.8 ± 0.6	1.1 ± 0.2	< 0.001

HOMA-IR	47.2 ± 4.9	5.0 ± 0.7	< 0.001

Serum cholesterol (mg/dl)	228 ± 5	89 ± 4	< 0.001

### Mast cells were distributed differentially in abdominal fat depots

**Figure 2:**
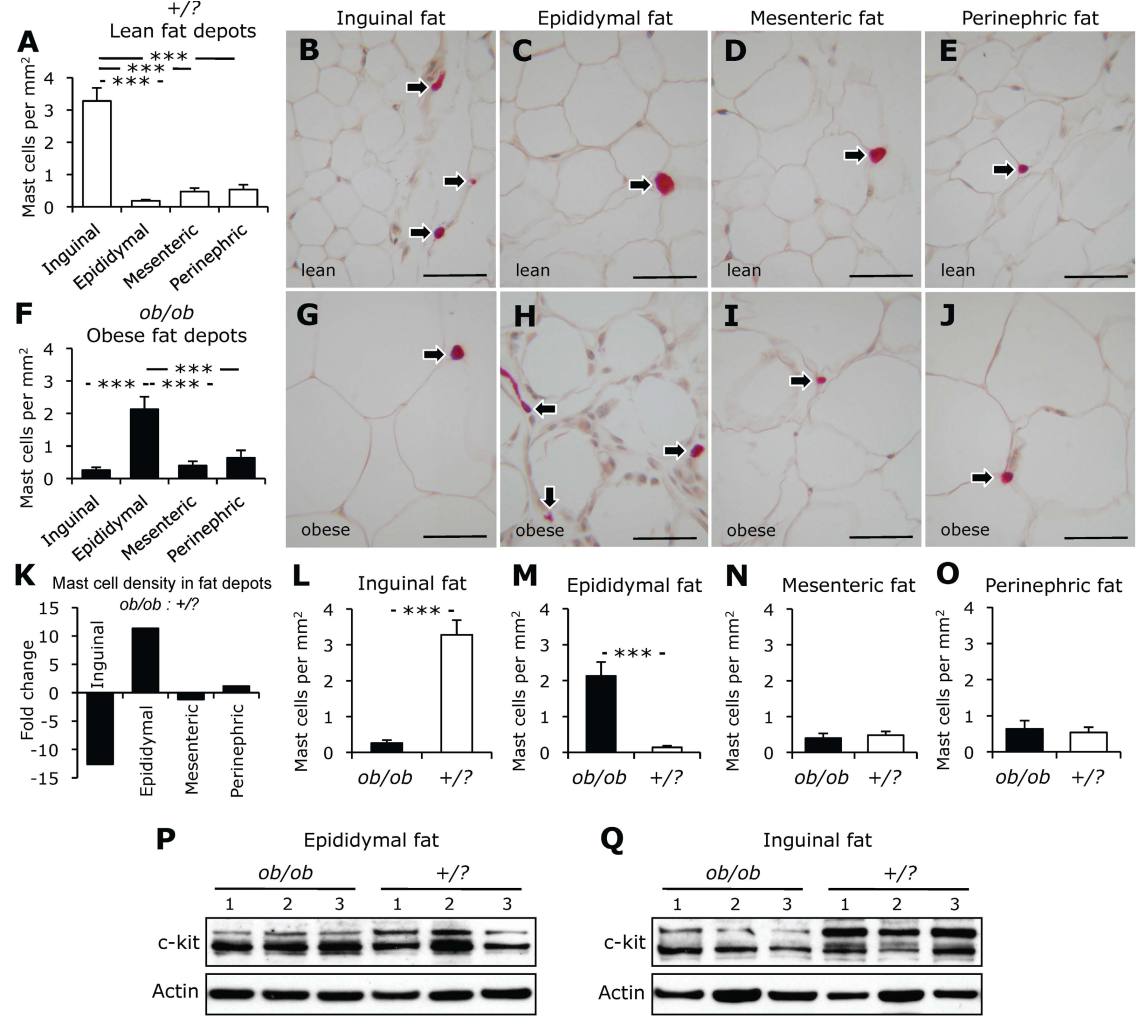
**Mast cells in abdominal fat depots**. The density of mast cells in epididymal, mesenteric, perinephric, and inguinal (subcutaneous) fat depots from control (*+/?*, n = 10, white bars) and *ob/ob *mice (n = 10, black bars) are shown (A-J). Leptin deficiency-induced obesity was accompanied by a divergent change in the density of mast cells in inguinal and epididymal adipose tissues (K-O). Photomicrograhs demonstrate mast cells (arrows) stained for chloroacetate esterase. CD117/c-kit protein expression in epididymal and inguinal fat depots from ob/ob and control mice are also shown (P, Q). Scale bars are 50 μm. ***P < 0.001.

Mast cells were present in abdominal fat depots of *ob/ob *and control mice (Figure 2). The density of mast cells, however, differed from one fat depot to another. In control mice, the density of mast cells was higher in subcutaneous than in visceral fat depots (Figures 2A-E). Moreover, the density of mast cells was 2.5-fold higher in mesenteric and perinephric than in epididymal fat in control mice (Figure 2A). In *ob/ob *mice, however, the density of mast cells was higher in epididymal fat than in inguinal, mesenteric, and perinephric fat depots (Figures 2F-J).

### Leptin deficiency led to a divergent change in the density of mast cells in inguinal and epididymal fat

Leptin deficiency-induced obesity was accompanied by a 20-fold increase in the density of mast cells in epididymal fat, but a 13-fold decrease in inguinal fat (Figures 2K-M). No statistically significant difference in the density of mast cells in mesenteric and perinephric fat depots between *ob/ob *and control mice was observed (Figures 2N, O). To confirm divergent alteration in the density of mast cells in epididymal vs. inguinal adipose tissue, tissue homogenates were subjected to immunoblotting for CD117/c-kit, a transmembrane tyrosine kinase receptor highly expressed in mast cells. Consistent with increased mast cell density in epididymal fat with leptin deficiency, Western blot analysis demonstrated increased CD117/c-kit expression (Figure 2P). A decline in mast cell density in inguinal fat with leptin deficiency was accompanied by diminished CD117/c-kit protein expression (Figure 2Q).

### The proportion of mast cells immunoreactive for TNF-α was higher in epididymal than in inguinal fat

**Figure 3:**
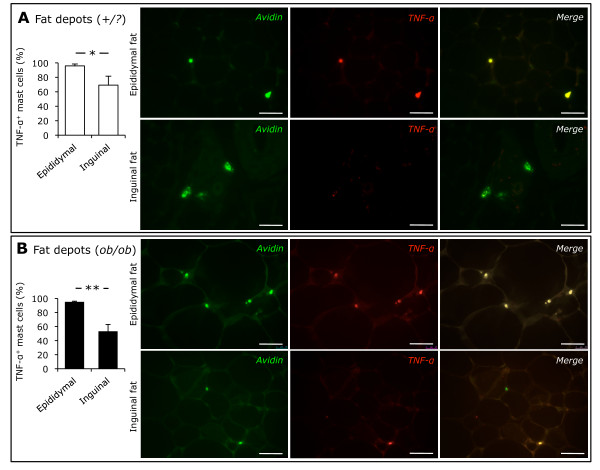
**TNF-α immunoreactivity in adipose tissue mast cells**. Immunofluorescence microscopy demonstrated mast cells labeled with FITC-conjugated avidin (green) and Alexa Fluor 594-labeled TNF-α (red) in epididymal and inguinal adipose tissues from control (*+/?*, n = 5, white bars, panel A) and *ob/ob *(n = 5, black bars, panel B) mice. Scale bars are 50 μm. * *P *< 0.05, ** *P *< 0.01.

We have previously shown that adipose tissue mast cells may be immunoreactive for TNF-α (11). Considering biological differences between subcutaneous and visceral fat, we tested whether the prevalence of mast cells immunoreactive for TNF-α was different between inguinal and epididymal fat. We found that 96% of mast cells in epididymal fat from control mice were immunoreactive for TNF-α. However, only 69% of those in inguinal fat showed TNF-α immunoreactivity (Figure 3A). In *ob/ob *mice, TNF-α immunoreactivity was also observed in the vast majority of mast cells (95%) in epididymal fat, while only 53% of mast cells in inguinal fat were immunoreactive for TNF-α (Figure 3B). Therefore, the proportion of mast cells immunoreactive for TNF-α was greater in visceral than in subcutaneous fat in both *ob/ob *and control mice.

### Mast cells were distributed differentially in abdominal lymph nodes

**Figure 4:**
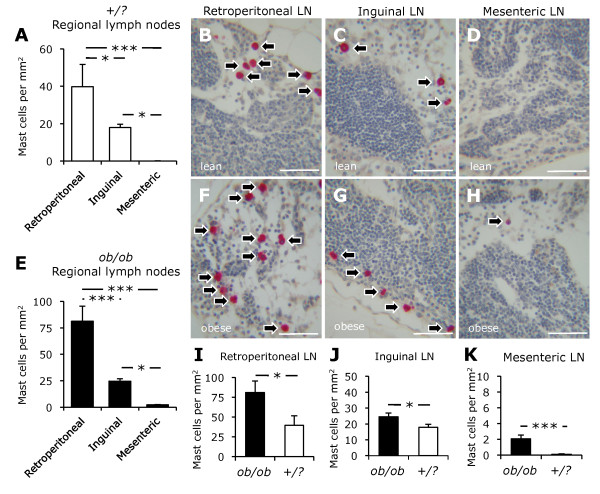
**Mast cells in abdominal lymph nodes**. The density of mast cells in retroperitoneal, mesenteric, and inguinal lymph nodes (LN) from control (*+/?*, n = 10, white bars) and *ob/ob *(n = 10, black bars) mice is shown (A-H). Note the differential distribution of mast cells in abdominal lymph nodes in both control and *ob/ob *mice (A, E). Leptin deficiency-induced obesity was accompanied by an increase in the density of mast cells in abdominal lymph nodes (I-K). Photomicrograhs demonstrate mast cells (arrows) stained for chloroacetate esterase. Scale bars are 50 μm. * *P *< 0.05, *** *P *< 0.001.

Tissue inflammation often leads to immune responses in draining lymph nodes. Here, we sought to determine the effects of leptin deficiency-induced obesity on the density of mast cells residing in abdominal lymph nodes. We found that mast cells populated retroperitoneal, mesenteric, and inguinal lymph nodes in both *ob/ob *and control mice (Figure 4). In control mice, the density of mast cells was significantly higher in retroperitoneal (40 ± 12 cells/mm^2^) than in inguinal (18 ± 2 cells/mm^2^) and mesenteric (0.1 ± 0.1 cells/mm^2^) lymph nodes (Figures 4A-D). Similarly, retroperitoneal lymph nodes had a higher density of mast cells (81 ± 14 cells/mm^2^) than inguinal (25 ± 2 cells/mm^2^) and mesenteric (2.1 ± 0.5 cells/mm^2^) lymph nodes in *ob/ob *mice (Figures 4E-H). In both *ob/ob *and control mice, the density of mast cells was higher in inguinal than in mesenteric lymph nodes.

### Leptin deficiency-induced obesity was accompanied by increased mast cell density in abdominal lymph nodes

Mast cells were more prevalent in retroperitoneal, mesenteric, and inguinal lymph nodes from *ob/ob *than those from control mice (Figures 4I-K). The density of mast cells in retroperitoneal lymph nodes was twofold higher in *ob/ob *than in control mice (Figure 4I). Moreover, mast cells were 40% more prevalent in inguinal lymph node from *ob/ob *mice than in that from control mice (Figure 4J). The density of mast cells in mesenteric lymph nodes was also higher in *ob/ob *than in control mice (Figure 4K).

### The density of mast cells in skeletal muscle, liver, spleen, and thymus was not affected by leptin deficiency

**Figure 5:**
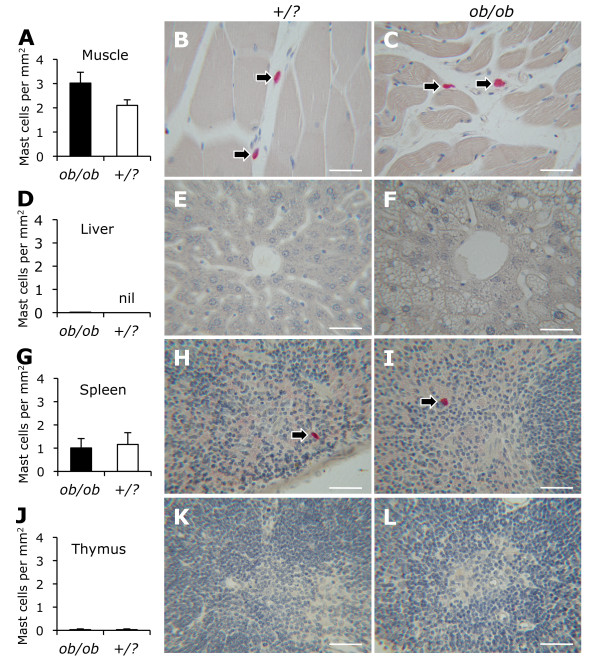
**Mast cells in skeletal muscle, liver, spleen, and thymus**. The density of mast cells in gastrocnemius muscle (A-C), liver (D-F), spleen (G-I), and thymus (J-L) from *ob/ob *(n = 10, black bars) and control (*+/?*, n = 10, white bars) mice is shown. Photomicrograhs demonstrate mast cells (arrows) stained for chloroacetate esterase. Scale bars are 50 μm.

There was no significant difference in the density of mast cells in the gastrocnemius muscle between *ob/ob *and control mice (3.0 ± 0.4 vs. 2.1 ± 0.2 cells/mm^2^) (Figure 5A). Mast cells were rare in the liver of both *ob/ob *(0.02 ± 0.02 cells/mm^2^) and control (0.00 ± 0.00 cells/mm^2^) mice (Figure 5B). The density of mast cells in the spleen of *ob/ob *mice (1.0 ± 0.4 cells/mm^2^) was similar to that of controls (1.2 ± 0.5 cells/mm^2^) (Figure 5C). Mast cells were rare in the thymus of both *ob/ob *(0.03 ± 0.03 cells/mm^2^) and control (0.03 ± 0.03 cells/mm^2^) mice (Figure 5D).

## Discussion

Here, we demonstrated that mast cells are distributed differentially in abdominal fat depots and lymph nodes in leptin-deficient obese mice. With respect to abdominal fat depots, leptin deficiency-induced obesity was accompanied by a substantial increase (20-fold) in the density of mast cells in epididymal fat, while a remarkable decrease (11-fold) in the density of mast cells in inguinal (subcutaneous) fat was observed. This divergent alteration in the density of mast cells was confirmed by CD117/c-kit protein expression analysis. Furthermore, the proportion of mast cells immunoreactive for TNF-α was significantly greater in epididymal than in inguinal fat. Leptin deficiency-induced obesity was associated with increased mast cells in abdominal lymph nodes. We found no significant difference in the density of mast cells in skeletal muscle, liver, spleen, and thymus between leptin deficient and control mice.

The structural and functional differences between fat depots in various anatomical locations have been the subject of much interest [12-14]. Since the discovery of adipose tissue inflammation in obesity and its impact on systemic insulin sensitivity, numerous studies have examined immune responses in fat depots in obese rodents and humans [11,17,18]. Our group recently reported that a high-fat diet (60% calories from fat) fed for 20 weeks led to an increase in mast cells in epididymal (90-fold), perinephric (24-fold), and mesenteric (7-fold) fat depots of 6-month-old C57BL/6 mice [11]. However, we found no significant difference in the density of mast cells in inguinal fat between diet-induced obese and control mice [11]. We believe that the differences observed between these two studies can be explained by at least three factors: 1) the duration of obesity; 2) the experimental diet; and 3) the proinflammatory properties of leptin, as discussed below.

Similar to other inflammatory diseases, adipose tissue inflammation in obesity is a dynamic pathophysiological process [18]. Neutrophils infiltrate visceral fat of mice within a week after high-fat feeding [19]. This is followed by macrophage accumulation and formation of crown-like structures [3,4]. Over time, adipose tissue inflammation in obesity is associated with collagen deposition and tissue fibrosis [18]. Thus, the type and severity of inflammatory changes in a specific fat depot is in part a function of the duration of obesity. Moreover, since fat depots have different propensity for obesity-associated inflammation, at any given time-point the severity of inflammation is different from one fat depot to another. In addition, the development of obesity and associated adipose tissue inflammation also depends on the type of diet consumed. We believe that a high-fat diet leads to more severe adipocyte injury and associated adipose tissue inflammation than the intake of larger quantities of a standard chow, as it is the case with leptin-deficient mice in the present study.

Pivotal in the regulation of energy homeostasis, metabolism, and neuroendocrine functions, leptin also plays an important role in innate and adaptive immune responses. Leptin can induce TNF-α and IL-6 production by monocytes [20]. Leptin can also augment the ability of macrophages to phagocytize pathogens [21]. For example, macrophages harvested from leptin-deficient mice showed reduced phagocytosis of bacteria [22]. Since leptin is required for lymphopoiesis, leptin receptor-deficient mice have fewer circulating B- and CD4^+ ^T-lymphocytes and are unable to correct irradiation-induced lymphopenia [23]. By stimulating IL-2 and IFN-γ and suppressing IL-4 production, Leptin may favor proinflammatory T-lymphocyte responses as well [24]. Leptin is also found to be important in the development and activation of natural killer cells [25]. Thus, a lower degree of adipose tissue inflammation in *ob/ob *mice can also be accounted for by diminished innate and adaptive immune responses due to leptin deficiency.

A proinflammatory cytokine with diverse biological effects, TNF-α plays a critical role in the pathogenesis of obesity-linked insulin resistance [2]. Mast cells may contain preformed TNF-α [26]. Moreover, upon proper stimulation, TNF-α protein and gene expression can be upregulated in mast cells [26]. We have previously shown that mast cells in the epididymal fat of diet-induced obese mice store and secrete TNF-α [11]. Here we showed that the majority of mast cells in epididymal and subcutaneous adipose tissue of both leptin-deficient obese and control mice are immunoreactive for TNF-α. However, we found that the proportion of mast cells immunoreactive for TNF-α was significantly higher in epididymal than in subcutaneous adipose tissue. Although other inflammatory and non-inflammatory cells in adipose tissue are capable of expressing TNF-α, a lower proportion of mast cells immunoreactive for TNF-α might be an important mechanism for the resilience of subcutaneous adipose tissue to metabolic challenges and consequent adipose tissue inflammation in obesity.

Lymph nodes are strategically located lymphoid tissues where innate immune responses can result in the induction of adaptive immunity [12]. Macrophages, B- and T-lymphocytes form the bulk of a lymph node. Although lymph nodes are involved in most immune responses, little is known about immunological reactions occurring in regional lymph nodes draining fat depots in obesity. A recent report indicated that increased T-lymphocyte activation and apoptosis was associated with decreased numbers of CD4^+ ^and CD8^+ ^T-lymphocytes in mesenteric lymph nodes of diet-induced obese mice [27]. Moreover, decreased proportions of CD8^+ ^T-lymphocytes and higher proportions of helper T-cell subsets in mesenteric lymph nodes of genetically obese rats compared to lean controls [28].

Mast cells are scattered in the cortical and medullary sinuses of murine lymph nodes [29]. While much is known about immune responses that take place in a lymph node, the immunological functions of mast cells in a lymph node remain largely elusive. It has been shown that mast cells residing in a lymph node can facilitate recruitment of T-lymphocytes by secreting chemokines such as macrophage inflammatory protein-1B [30]. Furthermore, mast cells in inflamed tissues can cause enlargement and activation of a draining lymph node by releasing TNF-α that is transported to draining lymph nodes [31]. In addition, it has been shown that dermal mast cells can migrate to draining lymph nodes and induce adaptive immune responses [30,32]. The present study is the first to show an increase in the density of mast cells in regional lymph nodes draining abdominal fat depots in obesity. The increased numbers of mast cells in the abdominal lymph nodes could be due to 1) increased mitotic activity of resident mast cells, 2) enhanced recruitment of precursor cells, 3) immigration from inflamed adipose tissue, and/or 4) decreased emigration. Although the contributions of mast cells to the activation of the immune system are widely recognized, under certain conditions mast cells can suppress immune responses [33]. Thus, further studies are required to delineate the role of mast cells in the lymph nodes draining inflamed fat depots in obesity.

The present study describes the effects of leptin deficiency-induced obesity on the distribution of mast cells in subcutaneous and visceral fat depots and regional lymph nodes. Important differences were noted between subcutaneous and epididymal fat depots. Increased density of mast cells in abdominal lymph nodes in obesity represents an anatomical link between adipose tissue inflammation and adaptive immune responses in lymph nodes. This work will stimulate design and implementation of mechanistic studies addressing immunological functions of mast cells in adipose and secondary lymphoid tissues in obesity.

## Abbreviations

TNF-α: tumor necrosis factor-α; LN: lymph node.

## Competing interests

The authors declare that they have no competing interests.

## Authors' contributions

MMA, BN, ECW, KBJ, GG, LMO researched data. MMA, JR, ND reviewed/edited manuscript. AN designed experiments, researched data, and wrote manuscript. All authors' read and approved the final manuscript.
